# Petrol Poisoning by Inhalation

**Published:** 1910-01-01

**Authors:** 


### PETROL POISONING BY INHALATION.

That the fumes of petrol are not good for us has probably been the feeling of many, though cases of anæmia and chronic ill-health of the pasty-face type are much commoner than are those in which the symptoms have been acute. The following instance of acute petrol poisoning, however, occurred under the observation of Major B. W. Longhurst, of the Royal Army Medical Corps:—

About 6 a.m. on April 20, 1909, Mrs. X was washing her head with petrol, which had been recommended by one of her relations as "a good thing" for the hair; she poured the petrol into a wash-hand basin, and then, bending over, bathed her hair in it. While this performance was taking place she became giddy, staggered about the room, and fell on her bed apparently unconscious. Her husband immediately sent for advice and explained what had occurred. The odour of the petrol in the house and in the bedroom was overpowering. Mrs. X was lying on her bed in an almost unconscious condition, the pupils were widely dilated, the skin was cold and clammy, the breathing stertorous, and the pulse small and almost imperceptible. Her face was dusky in colour and swollen to a remarkable degree.

She was forthwith removed to another room, and placed in front of open windows in a current of fresh air. Signs of improvement showed themselves immediately; she gradually regained consciousness; violent clonic spasms with shouting and screaming occurred, symptoms practically identical with those so often seen during the recovery from anæsthesia by nitrous oxide gas and ether. Soon after this the patient passed into a condition of collapse and shivered violently; she was placed on a couch, covered with blankets, and a little whisky and milk were administered; she remained quiet for about two hours, her temperature was subnormal but the pulse-rate was over 160 (varying from 160 to 200), violent tachycardia being present. As the patient recovered consciousness it was found that she was almost unable to move her arms and legs. A mixture containing digitalis was prescribed, and the palpitation had much improved towards evening.

On the second day palpitation was still present, the pulse-rate being over 100, but the patient did not complain of the thumping of the heart which was so distressing on the first day; the movements of the limbs were still very feeble, and she complained of stiffness of the fingers, also contracture of the left arm, with a peculiar tingling sensation amounting to actual pain. She was now able to take solid food and to go out for a drive in the evening. On the third day she had practically recovered, but still complained of the peculiar symptoms in the left arm, which remained for several days and then gradually disappeared under the influence of digitalis. Cases of this kind have proved fatal, and it is probable that this case might have been fatal had not the patient been removed to another room in time.

